# Using syndromic surveillance for unintentional and undetermined intent drowning surveillance in a large metropolitan area

**DOI:** 10.1186/s40621-024-00529-x

**Published:** 2024-09-26

**Authors:** Rohit P. Shenoi, Briana Moreland, Jennifer L. Jones, Nicholas Peoples, Elizabeth A. Camp, Ned Levine

**Affiliations:** 1 Division of Emergency Medicine, Department of Pediatrics, Texas Children’s Hospital and Baylor College of Medicine, 6621 Fannin St., Suite A 2210, Houston, TX 77030 USA; 2 Division of Injury Prevention, National Center for Injury Prevention and Control, Centers for Disease Control and Prevention, 4770 Buford Hwy, Atlanta, 30341 USA; 3 Student Services, Baylor College of Medicine, 1 Baylor Plaza, Houston, TX 77030 USA; 4 Ned Levine and Associates, Houston, TX 77025 USA

**Keywords:** Syndromic surveillance, Drowning burden, Drowning trends, Accuracy

## Abstract

**Introduction:**

A drowning definition is available for use with National Syndromic Surveillance Program (NSSP) data. However, its accuracy in capturing drowning emergency department and urgent care visits at the regional level is unknown. We tested the ability of the syndromic surveillance (SS) definition in capturing unintentional and undetermined intent drowning (UUID) and describe UUID SS visit trends in a large metropolitan area.

**Methods:**

We applied the drowning definition to NSSP data from 2016 to 2022 for the 8-county metropolitan Houston region. We queried the dataset for UUID ICD-10-CM codes and manually reviewed the chief complaint (CC) and discharge diagnosis (DD) for SS visits. True-positives were calculated by dividing the number of UUID cases identified by UUID ICD-10-CM codes and CC/DD review by the total visits captured by the SS definition. Demographics and trends of UUID visits were calculated from 2018 to 2022 due to limited data from 2016 to 2017 in NSSP.

**Results:**

2,759 visits were captured by the SS definition. After case review, 2,019 (73.2%) had ICD-10-CM drowning codes of any intent; and 2,015 of those (99.8%) were classified as UUID. Of the remaining 740 cases with no ICD-10-CM codes that were pulled by the SS definition, 690 (93.2%) had a CC/DD diagnosis of drowning/submersion/underwater related to aquatic exposure. Taken together, 2,705 (98.0%) were true-positive UUID visits based on the SS drowning definition.. Children (aged < 18 years) constituted 79% of UUID visits. Black, White and Asian/Pacific Islander persons comprised 17%, 60% and 4% of UUID visits respectively. Rates of UUID visits were lowest in 2020.

**Conclusion:**

Syndromic surveillance is a novel and accurate method to conduct real-time drowning surveillance in a large metropolitan region.

**Supplementary Information:**

The online version contains supplementary material available at 10.1186/s40621-024-00529-x.

## Introduction

Drowning is the leading cause of death among children aged 1–4 years in the United States and the second leading cause of unintentional injury death for children aged 5–14 years of age (Centers for Disease Control and Prevention 2023). There are approximately 800 fatal unintentional drownings involving US children aged 0–17 years each year in the United States (Centers for Disease Control and Prevention 2023). Drowning is preventable. Real time surveillance of drowning at the local and regional level can inform drowning prevention strategies. The National Syndromic Surveillance Program (NSSP) is a collaboration between the Centers for Disease Control and Prevention (CDC), local and state health departments, and health care facilities to collect, analyze, and share electronic patient encounter data received from EDs, urgent and ambulatory care centers, inpatient health care settings, and laboratories (The National Syndromic Surveillance Program (NSSP) 2024). The CDC’s National Center for Injury Prevention and Control adapted a drowning syndromic surveillance definition from Austin Public Health to use in NSSP data to provide timely information about drowning ED visits (The National Syndromic Surveillance Program (NSSP) 2024; Yoon et al. 2017; Update 2019). However, the ability of the “CDC unintentional drowning v1” syndrome definition in capturing both drowning emergency department (ED) and urgent care (UC) visits (collectively termed Syndromic Surveillance (SS) visits) in NSSP, and its use in drowning surveillance at the regional level, has not been described.

The aim of this study was to determine the percentages of true-positive unintentional and undetermined intent drowning (UUID) cases for all ages in a large metropolitan area based on all cases captured by the “CDC unintentional drowning v1” definition. Secondary aims were to use syndromic surveillance (SS) data to describe the burden and injury trends of UUID rates overall in the metropolitan Houston region and to compare demographic trends of SS data with National Vital Statistics System (NVSS) mortality data between 2018 and 2022.

## Methods

This cross-sectional study used NSSP data for the 8-county metropolitan Houston region from January 1, 2016, to December 31, 2022, from the City of Houston Health Department (HHD) Electronic Surveillance System Early Notification of Community-based Epidemics (ESSENCE) platform. The HHD-ESSENCE platform includes data from 128 of 143 (90%) healthcare facilities across the Public Health Region 6/5 South in Texas who agree to provide data to the City of Houston Health Department. Cases were pulled from the HHD-ESSENCE platform based on the patient’s county of residence being one of the following eight Texas counties: Brazoria, Chambers, Fort Bend, Galveston, Harris, Liberty, Montgomery, and Waller. These counties constitute Texas Public Health Region 6/5S. The population of the region was 7,092,073 in 2020. Children aged 0–17 years comprised 26.2% of the population and non-Hispanic White persons-33.9%, Black persons-18.5%, Asian persons 8.3% and American Indian/Alaska Native/Pacific Islander persons 1.1% of the population. Persons of Hispanic ethnicity comprised 38.8% of the population (US 2023).

### National syndromic surveillance program

The NSSP collects data from over 78% of EDs across the country. Electronic health data are transmitted to a shared platform called BioSense, and public health agencies, including the CDC and local and state health departments, can use analytic tools on the platform to analyze data received as early as 24 h after a patient’s visit for detection, characterization, monitoring, and response to events of public health concern (The National Syndromic Surveillance Program (NSSP) 2024). Visit information captured by the NSSP can include free-text chief complaint, discharge diagnosis codes, and some patient demographic information, such as age and sex. Diagnostic information is collected using the International Classification of Diseases, 9th Revision, Clinical Modification (ICD-9-CM), International Classification of Diseases, 10th Revision, Clinical Modification (ICD-10-CM), and Systematized Nomenclature of Medicine codes (The National Syndromic Surveillance Program (NSSP) 2024; Yoon et al. 2017).

In collaboration with several health departments, CDC adapted a drowning definition from Austin Public Health which is available in the ESSENCE platform (Moreland et al. 2024). The “CDC unintentional drowning v1” definition queries patient discharge diagnosis and chief complaint information to identify initial unintentional drowning encounters. The syndrome definition includes relevant ICD-10-CM codes (T75.1, V90, V92, W16 (with 6th character = 1 except 16.4 and 16.9 where 5th character = 1), W22.041, W65-W74, codes only included when 7th character = A or missing) (Moreland et al. 2024). Additionally, undetermined drowning encounters (Y21) were added to the definition for this analysis (Additional File 1). In addition to discharge diagnosis codes, the definition relies on the chief complaint inclusion (e.g., “drown”, “underwater”, “submerge” and respiratory symptoms related to contact with a body of water) and exclusion terms (e.g., “drown self”, “attempt to drown”, and “feel or sound like drowning”) to further identify drowning–related ED and urgent care visits and decrease false positives (Moreland et al. 2024). The HHD utilized the CDC unintentional drowning v1 definition (Moreland et al. 2024) to query the ESSENCE platform and obtain an output of all cases of UUID for the geographic area of interest. This was shared with the researchers through a data use agreement.

### Validation of the syndrome definition in the Houston Metropolitan area

Visits captured by the “CDC unintentional drowning v1” definition from 2016 to 2022 were queried using ICD-10-CM codes for UUID cases using SAS version 9.4 and manually reviewing the text describing the chief complaint and discharge diagnosis for ED and UC visits. We selected cases with a chief complaint or discharge diagnosis text indicating “drowning” or “submersion” or “underwater” or “inhaled water” or “swallowed water” in relation to contact with a body of water and/or specific misspellings. We excluded cases where the chief complaint/discharge diagnosis did not indicate drowning (Moreland et al. 2024).

### Analysis

To calculate the percentage of true-positives, we divided the number of UUID cases based on ICD-10-CM codes and manual review of chief complaint/discharge diagnosis by the total visits captured by the syndromic definition for drowning from 2016 to 2022. The City of Houston instituted syndromic surveillance in 2016 but it was not fully operational until 2018 leading to a likely underestimation of UUID visits in 2016 and 2017. Due to the incomplete syndromic surveillance data available in 2016 and 2017, we limited the UUID trend analysis to 2018 onward. We calculated the percentage of all SS visits that were related to drowning from 2018 to 2022 overall and by age (all ages, 0–17 years), Hispanic ethnicity and race (Black, White, Asian/Pacific Islander, and other race). “Other race” was defined as American Indian or Alaska Native and other race not previously specified due to small counts.

Annual drowning rates per 100,000 SS visits were calculated as the number of SS visits for UUID divided by the total number of SS visits and multiplied by 100,000 for the period 2018–2022. SS visits consist of non-fatal and some fatal data and are useful for real-time surveillance. However, deaths occurring outside of the ED are not captured. NVSS data can be used to analyze all deaths. We do not know if demographic trends in fatal drowning are the same as demographic trends in nonfatal drowning. Therefore, we also compared demographics between UUID SS visits and NVSS data between 2018 and 2021 (2021 was the most recent full year of data available for NVSS at the time of analysis) for the 8-county Houston Metropolitan area (Centers for Disease Control and Prevention 2023). Fatal drowning cases in NVSS were those with ICD-10 codes: V90, V92, W65-W74. Statistical demographic comparisons were made for SS visits over time and SS visits vs. UUID cases. Since accuracy of capturing all unintentional drowning cases is a consideration for this study, unknown values were included in the analysis. Categorical data were analyzed using the Pearson Chi-Square test. A p-value < 0.05 was considered statistically significant. This study received institutional review board approval from the Baylor College of Medicine (protocol H-51536).

### Patient and public involvement

The study did not include patient and public input since the data were retrospectively collected. Research questions were developed based on existing gaps in drowning surveillance. Patients and the public were not involved in study design or recruitment. The study results (injury burden and trends) will be disseminated to the community after publication.

## Results

**Fig. 1:**
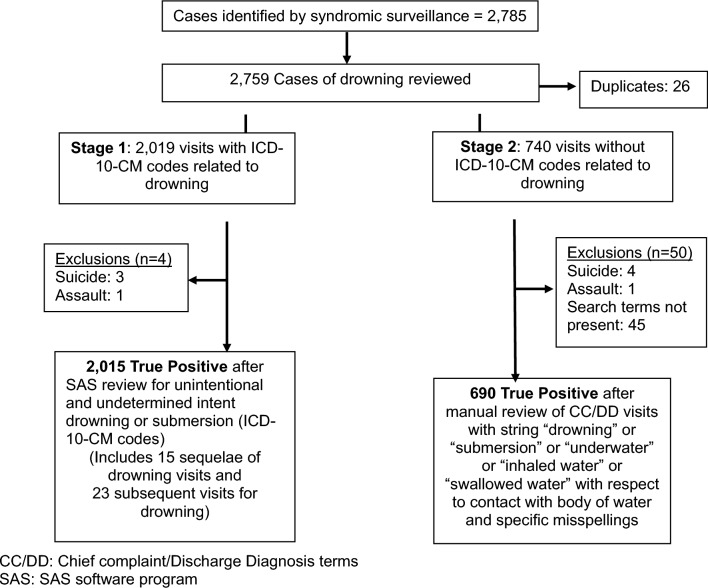
Analysis of drowning visits based on syndromic surveillance definition for unintentional/undetermined intent drowning for Houston metropolitan area (2016–2022)

During 2016–2022, there were 2,759 visits for UUID identified through SS. There were 2,019 cases (73.2%) with ICD-10-CM codes for drowning; of these 2,015 (99.8%) were classified as UUID (n = 4 intentional drowning cases). Of the remaining 740 cases with no ICD-10-CM codes, 690 (93.2%) were classified as UUID cases given chief complaint or discharge diagnosis text indicating “drowning” or “submersion” or “underwater” or “inhaled water” or “swallowed water” in relation to contact with a body of water and/or specific misspellings (Fig. 1). Taken together, among all 2,759 visits classified as drowning based on the SS definition, there were 2,705 (98.0%) cases classified as Yes (true-positive) UUID and 54 (2.0%) classified as No (exclusions) (Table 1).

**Table 1:** Results of visit reviews by coding stage for unintentional/undetermined intent drowning for Houston metropolitan area (2016–2022)

	Stage 1^a^	Stage 2^b^	Totals
Visits for Unintentional or Undetermined Drowning (UUID)	Visits with ICD-10-CM codes for UUID n = 2,019 (100%)	Visits with “submersion” or “drowning” or “underwater” or “swallowing water” or “inhaling water” with respect to contact with body of water and without ICD-10-CM codes for UUID n = 740 (100%)	All Visits 2,759 (100%)
Yes (True Positive)	2,015 (99.8%)	690 (93.2%)	2,705 (98.0%)
No (Exclusions)	4 (0.2%)	50 (6.8%)	54 (2.0%)

Houston Health Department, National Syndromic Surveillance Program

ICD-10-CM codes for UUID: T75.1XXA, W65-W74, V90, V92, W16 (with 6th character = 1 except 16.4 and 16.9 where 5th character = 1), W22.041, Y21 and excluding X71 and X92

^a^Cases captured by the drowning syndromic definition identified as UUID from ICD-10-CM codes

^b^Cases captured by the drowning syndromic definition that did not have UUID related ICD-10-CM codes

There were 24,742,818 (ED: 23,870,676; UCC: 872,142) SS visits captured by the Syndromic Surveillance Program for metropolitan Houston between January 1, 2018, to December 31, 2022. The number of UUID visits to the ED and UCC were 2,537 and 155 respectively for the same time period. Of the total 2,692 SS UUID visits for 2018–2022, there were 2,116 (78.6%) visits by children aged 0–17 years, 1,524 (56.6%) visits by males, 964 (35.8%) visits by persons of Hispanic ethnicity. The SS visits by race were as follows: Black persons: 447 (16.6%); White persons: 1,621 (60.2%); Asian/Pacific Islander persons: 112 (4.2%) and persons of other races: 421 (15.6%) during 2018–2022 (Table 2).

**Table 2:** Demographic characteristics of Syndromic Surveillance Unintentional or Undetermined Intent Drowning visits in Houston metropolitan area (2018–2022)

	Year					Total n (%) N = 2,692 (100)
	2018 n (%) n = 393(14.6)	2019 n (%) n = 624(23.2)	2020 n (%) n = 412(15.3)	2021 n (%) n = 626(23.2)	2022 n (%) n = 637(23.7)	
Male	208 (52.9)	359 (57.5)	245 (59.5)	365 (58.3)	347 (54.5)	1,524 (56.6)
Female	185 (47.1)	265 (42.5)	167 (40.5)	259 (41.4)	286 (44.9)	1,162 (43.2)
Unknown	0 (0.0)	0 (0.0)	0 (0.0)	2 (0.3)	4 (0.6)	6 (0.2)
Children 0–17 years	357 (90.8)	517 (82.9)	293 (71.1)	456 (72.8)	493 (77.4)	2,116 (78.6)
Adults	33 (8.4)	106 (16.9)	119 (28.9)	163 (26.1)	140 (22.0)	561 (20.8)
Unknown	3 (0.8)	1 (0.2)	0 (0.0)	7 (1.1)	4 (0.6)	15(0.6)
Race						
Black	47 (12.0)	89 (14.3)	56 (13.6)	115 (18.4)	140 (22.0)	447 (16.6)
White	227 (57.8)	396 (63.5)	279 (67.7)	378 (60.4)	341 (53.5)	1,621 (60.2)
Asian/Pacific Islander	10 (2.5)	24 (3.8)	14 (3.4)	30 (4.8)	34 (5.3)	112 (4.2)
Other Race^a^	64 (16.3)	95 (15.2)	61(14.8)	92 (14.7)	109 (17.1)	421 (15.6)
Unknown	45 (11.5)	20 (3.2)	2 (0.5)	11 (1.8)	13 (2.0)	91 (3.4)
Hispanic Ethnicity	130 (33.1)	244 (39.1)	150 (36.4)	218 (34.8)	222 (34.8)	964 (35.8)
Non-Hispanic	169 (43.0)	325 (52.1)	245 (59.5)	382 (61.0)	400 (62.8)	1,521 (56.5)
Unknown	94 (23.9)	55 (8.8)	17 (4.1)	26 (4.2)	15 (2.4)	207 (7.7)

Percentages may not total 100% due to rounding

Houston Health Department, National Syndromic Surveillance Program

^a^Other Race includes American Indian or Alaska Native and other race not mentioned

SS visits for UUID had a higher proportion of males, children aged 0–17 years and White persons compared to overall emergency department and urgent care visits (Table 3). From 2018 to 2022, the UUID drowning rate per 100,000 SS visits was lowest during 2020 (Table 4).

**Table 3:** Demographic characteristics of Unintentional or Undetermined Intent Drowning (UUID) visits and overall emergency department and urgent care visits in Houston metropolitan area (2018–2022)

	SS UUID visits n (%) 2,692 (100.0)	Overall visits n (%) 24,742,818 (100.0)
Male Sex	1,524 (56.6)	11,091,432 (44.8)
Unknown/Unreported	6 (0.2)	6,682 (0.03)
Children 0–17 years	2,116 (78.6)	14,656,990 (59.2)
Unknown/Unreported	14 (0.5)	–
Race		
Black	447 (16.6)	4,179,237 (16.9)
White	1,621 (60.2)	13,616,812 (55.0)
Asian/Pacific Islander	112 (4.2)	1,088,759 (4.4)
Other Race^a^	421 (15.6)	3,162,453(12.8)
Unknown/Unreported	91 (3.9)	2,695,557(10.9)
Hispanic Ethnicity	964 (35.8)	9,296,591 (37.6)
Unknown/Unreported	207 (7.7)	3,082,494 (12.5)

Percentages may not add up to 100 due to rounding

Houston Health Department, National Syndromic Surveillance Program

UUID: Unintentional or Undetermined Intent Drowning; SS: Syndromic Surveillance

^a^Other Race includes American Indian or Alaska Native and other race not mentioned

**Table 4:** Annual syndromic surveillance and UUID visits in Houston metropolitan area (2018–2022)

	Year				
	2018	2019	2020	2021	2022
UUID visits	393	624	412	626	637
Syndromic Surveillance* Visits	3,419,708	5,444,854	4,415,226	5,628,169	5,834,861
UUID visits/100,000 visits	11.49**	11.46**	9.33	11.12**	10.92**

Houston Health Department, National Syndromic Surveillance Program

UUID: Unintentional or Undetermined Intent Drowning

^*^Syndromic Surveillance visits = Emergency Department and Urgent Care visits

^**^ Significant at p = 0.01 compared to 2020 as reference

Comparison of SS visits based on male sex, age group 0–17 years, race, and Hispanic ethnicity with NVSS mortality data for UUID in the metropolitan Houston region for the years 2018–2021 revealed that there was a higher percentage of children aged 0–17 years (79.0%) (p < 0.05) and persons of Hispanic ethnicity (36.1%) (p < 0.05) and fewer males (57.3%) (p < 0.05), Black persons (14.9%) and White persons (62.3%) (p < 0.05) compared to NVSS data (children aged 0–17: 23.6%; Hispanic persons: 24.0%; males: 74.3%; Black persons: 23.4%; White Persons: 68.8%) (Table 5).

**Table 5:** Comparison of demographic characteristics of Syndromic Surveillance Unintentional or Undetermined Intent Drowning visits with NVSS drowning fatality data for the Houston metropolitan area (2018–2021)

	UUID SS^1^ n (%) 2,055 (100.0)	NVSS^2^ n (%) 475 (100.0)	p-value
Male Sex	1,177 (57.3)	353 (74.3)	p < 0.05
Children 0–17 years	1,623 (79.0)	112 (23.6)	p < 0.05
Race*			
Black	307 (14.9)	111 (23.4)	p < 0.05
White	1,280 (62.3)	327 (68.8)	
Hispanic Ethnicity	742 (36.1)	114 (24.0)	p < 0.05

Houston Health Department, National Syndromic Surveillance Program

^1^SS: UUID Syndromic Surveillance visits 2018–2021 Houston Health Department, National Syndromic Surveillance Program

^2^Centers for Disease Control and Prevention, National Center for Health Statistics. National Vital Statistics System (NVSS), Mortality 2018–2021 on CDC WONDER Online Database

Note: NVSS includes ICD-10 codes: V90, V92, W65-W74

^*^Values for Native Hawaiian or Pacific Islander, American Indian or Alaska Native and other race are suppressed in NVSS due to low case counts

During 2018–2022, the UUID drowning rate for males (6.16/100,000 SS visits) was higher than for females (4.70/100,000 SS visits). The UUID drowning rate for non-Hispanics (6.15/100,000 SS visits) was higher than for Hispanics (3.90/100,000 SS visits). Similarly, the UUID drowning rate for persons with White or Asian race (7.00/100,000 SS visits) was two times higher than for persons who were not White or Asian (3.51/100,000 SS visits) (data not shown).

## Discussion

In our study we observed that SS is a novel and accurate method of conducting real-time drowning surveillance in a large metropolitan region. Overall, 98% of the cases identified through SS were true-positive UUID visits. SS can be utilized to track the burden and trends of unintentional and undetermined drowning injuries at the state and regional level. Our results indicate that drowning rates between 2018 and 2022 as a percentage of SS visits in the metropolitan Houston region have been relatively stable except for the year 2020. This lower rate for 2020 coincided with the onset of the COVID-19 pandemic when stay at home orders and social distancing were in place during March and April, 2020 in Texas (Orders and to Mitigate Spread of COVID-19 In Texas 2023). Overall, ED visits declined in the United States during this time in NSSP data, especially among children (Hartnett et al. 2020). In the Great Lakes region, while drownings were lower than the historical average early in the pandemic, they began to increase as stay-at-home orders were lifted (Houser and Vlodarchyk 2021). The lower number of drowning and SS visits in 2018 could relate to the beginning of the roll out of the SS system in metropolitan Houston when participation by member entities was not complete as indicated by notably lower overall, all cause SS visits for that year.

We also observed that the distribution of SS visits based on pediatric age group, race and Hispanic ethnicity are different from those due to fatal drowning. This has implications in drowning injury surveillance and prevention. The SS data are unable to differentiate fatal and non-fatal drowning. Analysis of medical examiner data is a more accurate method to establish fatal drowning burden. However, SS can fill an important gap in drowning epidemiology, especially if syndromic surveillance data are linked with fatality data. Currently, there is no method which can study overall non-fatal drowning at a regional or state level. Syndromic surveillance for drowning can be used to monitor regional burden and regional and national trends in UUID in real time to facilitate drowning prevention efforts. Drowning prevention requires a multilayered approach and includes close, attentive, and constant adult supervision, functioning safety barriers, the presence of lifeguards, CPR training, basic swimming and water safety skills training, and the use of life jackets (Denny et al. 2021).

When comparing NVSS data with SS data, adults, males, Black persons, and non-Hispanic persons were more commonly represented in fatal drowning data. Fatal drownings are not always transported to the ED. This may be related to a number of factors. For example, drowning among adults more commonly occurs in natural water (Ryan et al. 2023; Xu 2014; Clemens et al. 2021) which may result in longer extrication times and death being declared at the scene. Disparities in drowning rates among different racial and ethnic groups may reflect variations in cultural practices, exposure to aquatic bodies, socio-economic factors, access to prevention strategies such as swimming lessons, and water competency among different population groups (Irwin et al. 2009; Pharr et al. 2018). This is a complex issue which would benefit from further study.

Syndromic surveillance has several limitations. First, the syndrome definition used might underestimate or overestimate UUID SS visits because of variation in coding, reporting, and the availability of visit-level data between facilities or over time. Second, the World Health Organization and International Liaison Committee on Resuscitation defines drowning as “the process of experiencing respiratory impairment from submersion or immersion in a liquid” (Morgan et al. 2022; Beeck et al. 2005) which is what was used in the SS UUID definition. While this drowning syndromic surveillance definition is sufficient to define drowning from the public health preventative perspective, these data may not be helpful when assessing the efficacy of drowning resuscitation and treatment. For instance, we included visits where there were respiratory symptoms like coughing, choking, or gasping for air that were associated with a body of water. In some cases, it is unclear whether the airway was actually submerged. Third, it is difficult to determine the intent of drowning in SS data. ICD-10-CM codes are not included in all SS visits and even when ICD-10-CM codes are included in the discharge diagnosis field, not all codes are associated with a specific intent. For instance, the ICD-10-CM code T75.1 can be used to describe unintentional or intentional drowning. Fourth, SS data lacks consistent information on patient disposition and it is difficult to determine if drowning visits are fatal or nonfatal. In these cases, medical examiner data would be more helpful for analyzing fatal drowning. Also, details on the body of water where the drowning occurred are not complete. Fifth, duplicate cases may arise when patients are transferred between facilities. Finally, syndromic surveillance data only cover approximately 78% of EDs in the country and are not nationally representative (The National Syndromic Surveillance Program (NSSP) 2024). However, in our region 90% of health care facilities participate on ESSENCE.

There are also several limitations specific to this study. First, the completeness of data may have varied during the study period because of varying participation in the NSSP by health facilities. We accounted for these possible fluctuations by assessing monthly trends in UUID SS visits as a percentage of the total number of monthly SS visits. However, this indicator could be influenced by changes in the denominator or by differences in the characteristics and exposure to water of the patients visiting the participating facilities. Currently 90% of health care facilities in the region submit data to ESSENCE. We did not correct for the population. Second, the percentage of UUID may vary based on the prevalence of other causes of SS visits and this may not reflect true increases in UUID. Third, the results are for one region in the US and may not be generalizable to other areas with different aquatic bodies of water or in regions which do not use NSSP. Fourth, all drowning deaths in the Houston Metropolitan area were included in the NVSS data, but only health care facilities that provide information to the City of Houston Health department were included in the SS data which could partially contribute to the differences in demographic characteristics between NVSS and SS data. Finally, while we were able to determine the percentage of true-positives identified by the syndrome, we were unable to describe the number of cases that the syndrome may have missed. A review of hospital records would address this issue but would be logistically difficult to conduct at a regional level.

## Conclusion

Syndromic surveillance data are a novel source for drowning injury surveillance. Nearly all cases of UUID captured by SS in a large metropolitan area appeared to be accurately classified, indicating a very high degree of accuracy for the SS definition. Syndromic surveillance can be used by local injury prevention professionals to monitor drowning injuries and to inform preventative measures.

## Supplementary Information

**Additional file 1**. ICD -10 CM codes for Unintentional and Undetermined Intent Drowning.

## Data Availability

Data will be available upon conclusion of the grant period on October 1, 2026.

## Abbreviations

ED: Emergency Department
ESSENCE: Electronic Surveillance System Early Notification of Community-based Epidemics
HHD: Houston Health Department
NSSP: National Syndromic Surveillance Program
NVSS: National Vital Statistics System
SS: Syndromic Surveillance
UC: Urgent Care
UUID: Undetermined and Unintentional and undetermined intent drowning
