# Supplementary material for: Using syndromic surveillance for unintentional and undetermined intent drowning surveillance in a large metropolitan area
Source: Inj Epidemiol. 2024 Sep 26;11(Suppl 1):52. doi: 10.1186/s40621-024-00529-x (PMC11426074; doi:10.1186/s40621-024-00529-x)
Supplement: Supplementary file 1 — Additional file 1. ICD -10 CM codes for Unintentional and Undetermined Intent Drowning. [file 40621_2024_529_MOESM1_ESM.docx]

**Additional File 1: ICD -10 CM codes for Unintentional and Undetermined Intent Drowning**

**W65-W74 (Accidental drowning and submersion)**

W65 (Drowning and submersion while in bath-tub)

W66 (Drowning and submersion following fall into bath-tub)

W67 (Drowning and submersion while in swimming-pool)

W68 (Drowning and submersion following fall into swimming-pool)

W69 (Drowning and submersion while in natural water)

W70 (Drowning and submersion following fall into natural water)

W73 (Other specified drowning and submersion)

W74 (Unspecified drowning and submersion)

**V90 (Accident to watercraft causing drowning and submersion)**

V90.0 (Accident to watercraft causing drowning and submersion, merchant ship)

V90.1 (Accident to watercraft causing drowning and submersion, passenger ship, ferry-boat, or liner)

V90.2 (Accident to watercraft causing drowning and submersion, fishing boat)

V90.3 (Accident to watercraft causing drowning and submersion, other powered watercraft, hovercraft (on open water), or jet skis)

V90.4 (Accident to watercraft causing drowning and submersion, sailboat or yacht)

V90.5 (Accident to watercraft causing drowning and submersion, canoe or kayak)

V90.6 (Accident to watercraft causing drowning and submersion, inflatable craft (nonpowered))

V90.7 (Accident to watercraft causing drowning and submersion, water-skis)

V90.8 (Accident to watercraft causing drowning and submersion, other unpowered watercraft, surf-board, or windsurfer)

V90.9 (Accident to watercraft causing drowning and submersion, unspecified watercraft, boat NOS, ship NOS, or watercraft NOS)

**V92 (Water-transport-related drowning and submersion without accident to watercraft)**

V92.0 (Water-transport-related drowning and submersion without accident to watercraft, merchant ship)

V92.1 (Water-transport-related drowning and submersion without accident to watercraft, passenger ship, ferry-boat, or liner)

V92.2 (Water-transport-related drowning and submersion without accident to watercraft, fishing boat)

V92.3 (Water-transport-related drowning and submersion without accident to watercraft, other powered watercraft, hovercraft (on open water), or jet skis)

V92.4 (Water-transport-related drowning and submersion without accident to watercraft, sailboat or yacht)

V92.5 (Water-transport-related drowning and submersion without accident to watercraft, canoe or kayak)

V92.6 (Water-transport-related drowning and submersion without accident to watercraft, inflatable craft (nonpowered))

V92.7 (Water-transport-related drowning and submersion without accident to watercraft, water-skis)

V92.8 (Water-transport-related drowning and submersion without accident to watercraft, other unpowered watercraft, surf-board, or windsurfer)

V92.9 (Water-transport-related drowning and submersion without accident to watercraft, unspecified watercraft, boat NOS, ship NOS, or watercraft NOS)

**T75.1XXA (Unspecified effects of drowning and nonfatal submersion, initial encounter)**

**W22.041 (Striking against wall of swimming pool causing drowning and submersion)**

**All W16 with 6th character=1 (Except W16.4 and W16.9 with 5th character=1)**

W16.41 (fall into unspecified water causing drowning and submersion)

W16.91 (jumping and diving into unspecified water causing drowning and submersion)

**Y21 (Drowning and Submersion Undetermined Intent)**

Y21.0XXA (Drowning and submersion while in bathtub, undetermined intent, initial encounter)

Y21.1XXA (Drowning and submersion after fall into bathtub, undetermined intent, initial encounter)

Y21.3XXA (Drowning and submersion after fall into swimming pool, undetermined intent, initial encounter)

Y21.4XXA (Drowning and submersion in natural water, undetermined intent, initial encounter)

Y21.8XXA (Other drowning and submersion, undetermined intent, initial encounter)

Y21.9XXA (Unspecified drowning and submersion, undetermined intent, initial encounter)
